# Differential Impact of Natural Disasters on Mental Health Across Racial and Ethnic Groups

**DOI:** 10.1093/geroni/igaf122.3257

**Published:** 2025-12-31

**Authors:** S M Foysol Ahmed, Yeon Jin Choi

**Affiliations:** University of Kentucky, Lexington, Kentucky, United States; University of Kentucky, Lexington, Kentucky, United States

## Abstract

Natural disasters are increasing in both frequency and severity, affecting 224.1 million people globally each year. These events have profound consequences for public health and well-being, as exposure to natural disasters has been found to be associated with poor mental health outcomes. The adverse impact of natural disasters may vary across racial and ethnic groups due to their differences in socioeconomic status, trauma exposure, and access to healthcare. However, existing literature primarily focuses either on a single racial and ethnic group or broadly on affected populations, limiting our understanding of how natural disaster exposure affects mental health across diverse racial and ethnic groups. This study examines whether lifetime experience of natural disasters is associated with poor mental health and whether the impact differs across racial and ethnic groups, utilizing data from the 2010/2012 Health and Retirement Study (N = 13,817). A series of weighted ordinary least squares regression models were estimated, adjusting for sociodemographic characteristics. The results indicate that natural disaster exposure is associated with higher levels of depression and anxiety, with a greater impact observed among older Blacks (depression: β=.52, p<.001; anxiety: β=.19, p<.001) compared to older Whites (depression: β=.11, p<.05; anxiety: β=.07, p<.001). The association was not significant among older Hispanics (depression: -.20, p=.45; anxiety: .02, p=.82). Our findings align with previous research indicating greater vulnerability to natural disasters among racial and ethnic minorities. They underscore the need for tailored policy and intervention strategies for these communities, such as culturally informed mental health care and disaster preparedness and response plans.

